# Recycling solid waste to develop a novel and sustainable glass shielding for nuclear and radiation applications

**DOI:** 10.1038/s41598-026-46099-4

**Published:** 2026-04-02

**Authors:** Mohamed Mitwalli, Mohammed Humaid, Jehad Asad, Mohammad Hasan Abu Mhareb, Abdalrhman AboAlatta, Yasser Alajerami, Naji AlDahoudi, Abdullah Metebi, Sahr Alzhrani

**Affiliations:** 1 Interdisciplinary Research Center for Industrial Nuclear Energy (IRC-INE), King Fahd University of Petroleum and Minerals (KFUPM), Dhahran, 31261 Saudi Arabia; 2 Medical Imaging Department, Al Azhar University-Gaza, Gaza, 1277 Palestine; 3 Physics Department, Al Azhar University-Gaza, Gaza, 1277 Palestine; 4 Department of Physics, College of Science, Imam Abdulrahman Bin Faisal University, Dammam, 31441 Saudi Arabia; 5 Basic and Applied Scientific Research Center, Imam Abdulrahman Bin Faisal University, Dammam, 31441 Saudi Arabia; 6 Radiological Sciences Department, Taif University, Taif, Saudi Arabia; 7 Department of Physical Sciences/Physics Division, College of Science, Jazan University, Jazan, 45142 Saudi Arabia

**Keywords:** Industrial nuclear energy, Glass shielding, Mechanical properties, Recycling, Waste management, PbO, Engineering, Environmental sciences, Materials science, Physics

## Abstract

This study investigates the mechanical properties and multifunctional radiation shielding performance of a Novel Glass System (NGS) synthesized from recycled waste materials. NGS was produced from discarded lead-acid batteries and automotive glass, representing a sustainable and environmentally responsible method of glass manufacturing through the reuse of lead-free industrial waste. A thorough characterization was performed by density measurements, compositional analysis, and assessment of mechanical properties utilizing the Makishima–Mackenzie model. Structural features were examined via Fourier Transform Infrared Spectroscopy (FTIR). The mechanical performance of the NGS was significantly influenced by the PbO content, which was varied from 10 to 50 mol%. With the rise in PbO content, a significant decline in both Young’s modules (from 73.446 GPa at 0% PbO to 37.480 GPa at 50% PbO) and hardness (from 4.709 GPa to 3.141 GPa) was noted, demonstrating adjustable mechanical properties. The radiation shielding properties, evaluated by the Exposure Buildup Factor (EBF) and Energy Absorption Buildup Factor (EABF), demonstrated significant energy dependency with noticeable peaks at specific penetration depths. Moreover, the Specific Absorbed Fraction of Energy (SAFE) and Gamma-rays Transmission Factor (GTF) illustrated the efficacy of the NGS in attenuating gamma radiation throughout a wide energy range. The results highlight the dual capabilities of the designed glass system, which integrates sufficient mechanical strength with effective gamma-rays shielding. These findings proved NGS as a viable option for sustainable shielding materials, enhancing the circular economy and tackling significant issues in radiation safety and waste management.

## Introduction

Solid Waste Recycling (SWR) plays an increasingly critical role in addressing global challenges related to waste management, environmental protection, and resource sustainability^1^. By recovering and reintegrating waste-derived materials into production cycles, SWR reduces landfill burden and supports circular economy principles. Among the most pressing waste streams are Spent Lead-acid Batteries (SLB) and end-of-life Vehicle Glass (VG). Inadequate disposal of SLBs can release hazardous lead compounds, such as PbO, into ecosystems, where they bioaccumulate and provide significant threats to human health and animals^2–4^. Automotive glass, despite being chemically inert, accumulates in landfills due to its slow breakdown and restricted reuse options. Recycling these materials provides a twofold advantage: reducing environmental risks while reclaiming valuable components for sophisticated functional uses^5^.

SLB are widely used in automotive and industrial sectors and contain large quantities of lead oxide (PbO). Owing to its high atomic number and density, lead is exceptionally effective for attenuating ionizing radiation, particularly gamma-rays through photoelectric absorption and Compton scattering^2,3,6^. As a result, lead has historically served as a standard material for radiation shielding^7–9^. Nonetheless, metallic lead is mechanically pliable and exceedingly poisonous, presenting considerable environmental and occupational health issues^2,3,6^. Recycling lead from SLB and integrating it into a stable solid state within a glass matrix provides a safer and more sustainable option. Glass encapsulation efficiently immobilizes PbO, significantly diminishing leaching concerns and environmental exposure. This strategy converts hazardous waste into a high-value shielding component, consistent with waste-to-resource and sustainable materials frameworks^4,10–13^.

In parallel, waste vehicle glass primarily soda-lime silicate represents an abundant and underutilized secondary raw material. Although recycling is complicated by laminated polymers and large volumes, VG is attractive due to its availability and relatively low remelting temperature. Using crushed VG as a silica source reduces reliance on virgin raw materials and lowers melting energy consumption; each 10% increase in cullet content can reduce energy demand by approximately 2–3%^14–16^. Furthermore, redirecting VG from landfills mitigates greenhouse gas emissions linked to traditional glass manufacturing. Incorporating recovered PbO with waste VG offers a viable approach to sustainable, glass-based radiation shielding systems that merge excellent attenuation effectiveness with diminished environmental impact^17^.

Radiation shielding glasses are esteemed for their mechanical strength, chemical resistance, and clarity, along with efficient radiation attenuation. Conventional high-performance shielding glasses typically depend on costly high-purity heavy metal oxides or rare-earth dopants, which escalates production costs and restricts large-scale implementation^2,18–21^. This has stimulated increasing interest in shielding materials created from garbage. Prior research has shown that the inclusion of waste glass in building and shielding composites markedly improves gamma attenuation. For example, red-clay bricks with up to 50% waste glass demonstrated a decrease in the Half-Value Layer (HVL) from approximately 6.13 cm to around 4.62 cm for 662 keV gamma-rays, signifying significantly enhanced shielding efficacy^22,23^. Cement mortars containing micro- or nano-sized waste glass powders exhibited improved mechanical strength and decreased gamma transmission, with peak performance noted at around 30% nano-glass substitution^6,24^. These findings affirm that waste glass can actively participate in radiation attenuation rather than only functioning as an inert filler^25–27^.

Advanced glass systems infused with heavy metals or rare-earth oxides have exhibited superior shielding efficacy and radiation resilience. Ce/Sb/Mn-doped lead borate glasses preserved structural integrity following gamma doses of up to 120 kGy and shown enhanced chemical endurance as a result of radiation-induced structural compaction. In many instances, gamma irradiation promoted bond rearrangements that diminished lead leachability^28^. Monte Carlo models and experimental observations verified that these glasses maintained elevated mass attenuation coefficients and low half-value layers, equivalent to or surpassing conventional shields^25,29^. Borosilicate glasses produced from municipal solid waste ash and doped with CeO₂ and/or Gd₂O₃ exhibited minimal structural or optical degradation during 100 kGy irradiation, in addition to enhanced gamma shielding efficacy compared to commercial concretes^21,30^. Advancements have been realised in transparent shielding glasses, wherein Gd₂O₃–Fe₂O₃ co-doped borosilicate systems have attained concurrent enhancements in density, gamma attenuation, and optical transparency, facilitating applications like radiation-viewing windows^30,31^.

The current study advances the development of a unique, multifunctional Novel Glass System (NGS) by incorporating recycled PbO from spent lead batteries into a waste vehicle glass matrix. The compositions are expressed as (90 – x)W–10B₂O₃–xPbO (mol%), with W representing waste VG and x varying from 0 to 50 mol%. B₂O₃ is utilized as a glass forming and neutron absorber, enhancing the gamma shielding offered by PbO. The influence of recycled PbO content on mechanical properties, gamma and neutron shielding efficacy, and structural attributes is carefully examined. Furthermore, sustainability criteria are assessed, encompassing hazardous waste immobilization, diminished consumption of virgin raw materials, and prospective reductions in energy usage and carbon footprint. Prior research indicates that substituting natural resources with recycled glass in radiation shielding materials can lead to carbon emission reductions of 34–64%^27^. The proposed NGS is anticipated to have a reduced lifespan environmental impact compared to traditional lead-glass systems. This work seeks to showcase a high-performance, eco-friendly radiation shielding glass that concurrently tackles waste management, resource circularity, and radiological protection requirements.

## Sampling and analysis

Lead oxide (PbO) was produced from used lead-acid batteries by a two-step leaching and calcination process. This procedure entails the chemical conversion of lead-containing chemicals found in the wasted battery paste. Initially, a combination of sodium citrate and acetic acid served as leaching agents. These reactants enable the selective dissolution of diverse lead compounds, facilitating their transformation into lead citrate via complicated processes. In the subsequent stage, the resultant lead citrate precursor underwent thermal breakdown (calcination) in an air environment, producing high-purity lead oxide. This method efficiently extracts Pb from toxic battery waste while reducing environmental hazards^2,32^. Recycled glass (designated as R) was sourced from the windscreen glass of end-of-life Audi vehicles, notably the Sucursiv E2 model.

The glass debris was initially cleaned by hand to eliminate surface impurities, then subjected to mechanical grinding using a mortar and pestle until a fine, homogeneous powder was produced. The analysis of the recycled glass’s chemical composition is displayed in Table 1. This glass functioned as the silicate-dense matrix in the formulated shielding glass system^33^. Glass samples were prepared with the chemical composition of (90 – x) W – 10 B₂O₃ – x PbO, where W denotes the molar content of PbO, altered at six levels: 0, 10, 20, 30, 40, and 50 mol%. The samples are labelled RBPb0, RBPb10, RBPb20, RBPb30, RBPb40, and RBPb50, respectively. For each glass composition, a 15 g batch of powdered precursors was accurately measured using a high-precision analytical balance with an accuracy of ± 0.0001 g. The components were dry combined to obtain a homogeneous blend and thereafter put into porcelain crucibles for melting. The melting procedure was executed in an electric furnace at temperatures between 1050 °C and 1100 °C for a duration of 1 h.

**Table 1:** Discrimination of glass system installation.

Code of sample	Chemical compositions (Mole fraction)			Density (g/cm^3^)
	W	B_2_O_3_	PbO	
RBPb50	4	1	5	5.29
RBPb40	5	1	4	5.19
RBPb30	6	1	3	4.70
RBPb20	7	1	2	4.06
RBPb10	8	1	1	3.33
RBPb0	9	1	0	2.47

To enhance melt homogeneity, the mixture was stirred manually every 15 min during the melting process. Immediately following the melting stage, the molten glass was rapidly compressed between two stainless steel plates to form flat disc-shaped specimens and reduce residual thermal stresses. The samples were subsequently annealed at 400 °C for 3–4 h in a separate muffle furnace to alleviate internal tensions and enhance structural stability. After annealing, the samples were allowed to cool gradually to ambient temperature in the furnace to prevent thermal shock and cracking.

### Mechanical Properties

Makishima and Mackenzie^14,23,34–37^ proposed a theoretical model to determine the elastic moduli. These moduli can be calculated based on chemical composition, which depends on dissociation energy (Gt) and packing density (Vt):

1 $${V_t}=\frac{\rho }{M}\mathop \sum \limits_{i} {x_i}{V_i}$$

2 $${G_t}=\mathop \sum \limits_{i} {x_i}{G_i}$$

where, Vi and Gi denote packing factor and dissociation energy per unit volume, respectively. The previous equation can be used to calculate elastic modulus according to the following relations:

3 $${\mathrm{Young}}{\prime }{\text{s modulus }}({\mathrm{E}},{\text{ GPa}}):E=8.36{V_t}{G_t}$$

4 $${\text{Bulk modulus }}({\mathrm{B}},{\text{ GPa}}):B=10V_{t}^{2}{G_t}$$

5 $${\text{Shear modulus }}({\mathrm{S}},{\text{ GPa}}):S=\frac{{3B}}{{10.2{V_t} - 1}}$$

6 $${\text{Longitudinal modulus (L}},{\text{ GPa)}}:L=B+\frac{4}{3}S$$

while the following relations were used to calculate the Poisson ratio (σ) and microhardness (H, GPa):

7 $$\sigma =0.5 - \left( {\frac{1}{{7.2{V_t}}}} \right)$$

8 $$H=\frac{{\left( {1 - 2\sigma } \right)Y}}{{6\left( {1+\sigma } \right)}}$$

### FTIR analysis

The FTIR spectra were executed to characterize the functional regions and acquire knowledge about the various structural groups present in the borosilicate glass with Pb. Utilizing the (PerkinElmer Spectrum Two FTIR Spectrometer), the spectra were recorded at room temperature, covering the range of 4000 cm^−1^ to 450 cm^−1^, enabling the examination of the internal structure of the glass samples. The measurements were determined using the KBr pellet technique. By mixing powdered samples with KBr, pellets were prepared. Pellets are analyzed using an FTIR spectrum from Perkin Elmer.

### Shielding properties

#### Exposure and absorbance buildup factor

The G-P approach requires five distinct fitting parameters in the second stage to estimate the Buildup Factor (BF). The American Nuclear Society (ANS) publication is referred to determine the coefficient parameters for the NGS within the new composition^38^. This publication provides coefficient parameters for 23 elements and 25 standard photon energies. In cases where the ratio of the new system does not correspond to any of the 23 elements, we utilized the following logarithmic interpolation formula to obtain the values of the G-P fitting parameters.

9 $$P=\frac{{{F_1}\left( {log{Z_2} - log{Z_{eq}}} \right)+{F_2}\left( {log{Z_{eq}} - log{Z_1}} \right)}}{{log{Z_2} - log{Z_1}}}$$

Concerning BF the calculation of both F1 and F2 represent the values coefficients of G-P fitting corresponding to the elements as related atomic numbers Z1 and Z2, respectively, which are published by ANS publication^38^. BF is then determined for specific energy levels and their corresponding penetration depths up to 40 mean free paths (mfp) using the formulas (10–12):

10 $$B(E,~x)=1+\frac{{(b - 1)({K^x} - 1)}}{{K - 1}},{\text{ for}}\;\;K \ne {\mathrm{1}}$$

11 $$B(E,~x)=1+(b - 1)x~for~K=1$$

where,

12 $$K(E,~x)=c{x^a}+d~\frac{{\tanh \left( {\frac{x}{{{X_k}}} - 2} \right) - \tanh ( - 2)}}{{1 - {\mathrm{tanh}}( - 2)}},~for~x \leqslant 40~mfp$$

where $E,~{\mathrm{and}}~x,$ are the photon energy and the penetration depth (mfp) respectively. The other parameters like $\left( {c,~a,~d,~{x_k}} \right),$ denote the G-P fitting factors as explained in^38^.

#### Specific gamma-rays index (*Γ*)

The Specific gamma-rays index (*Γ*) is the amount of radiation exposure (R/hr) resulting from photons energy ($\:{E}_{\gamma\:}$) at an adequate distance^39^. The current experiment used 1 m free space to radiation source with radioactivity of 1 Ci and (*Γ*) is calculated using the formula (13).

13 $$\Gamma =657.68~X~{E_\gamma }\left( {\frac{{{\mu _{en}}}}{\rho }} \right)\left( {\frac{{R.{m^2}}}{{Ci.hr}}} \right)$$

The dosage (D) received in the investigated glass system, located at the field of study (m) from a radiation source with radioactivity (Bq) at a preset time (h), as well (D) can be computed based on the (Γ) values using the following formula (14):

14 $$D=\frac{{\Gamma At}}{{{r^2}}}$$

#### Specific absorbed fraction of the energy (SAFE)

The Energy Absorption Buildup Factor (EABF) data is employed to calculate the received dosage in a homogenized object. The Specific Absorbed Fraction of the Energy (SAFE) can be determined through a certain distance (x) between the source of radiation and the investigated sample^31,40–42^ by using an Eq. (15).

15 $$SAFE=\frac{{{\mu _{en}}\exp (\mu \chi )(EABF)}}{{4\pi {\chi ^2}\rho }}({{\mathrm{g}}^{ - {\mathrm{1}}}})$$

where ρ, x, and µ_en_ are the glass density, the free space between the source of radiation and the glass sample, and the linear absorption coefficient.

Finally, the SRIM code was used to calculate both the mass stopping power and the predicted range of proton and alpha particles^29,43–45^ SRIM code provides a Monte Carlo simulation base for estimating ion-stopping power in substances^18,47^.

## Results and discussion

### NGS structural

**Fig. 1:**
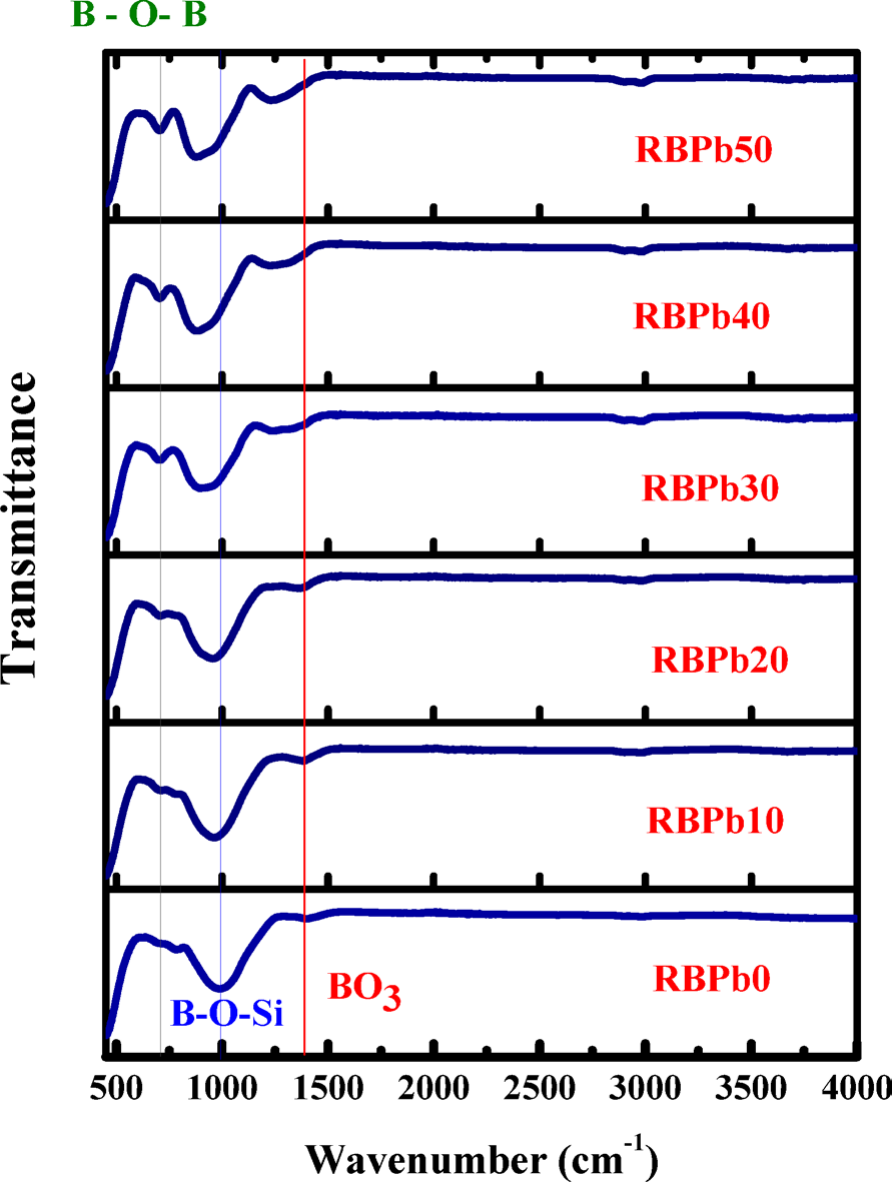
FTIR spectra of the fabricated glass.

**Fig. 2:**
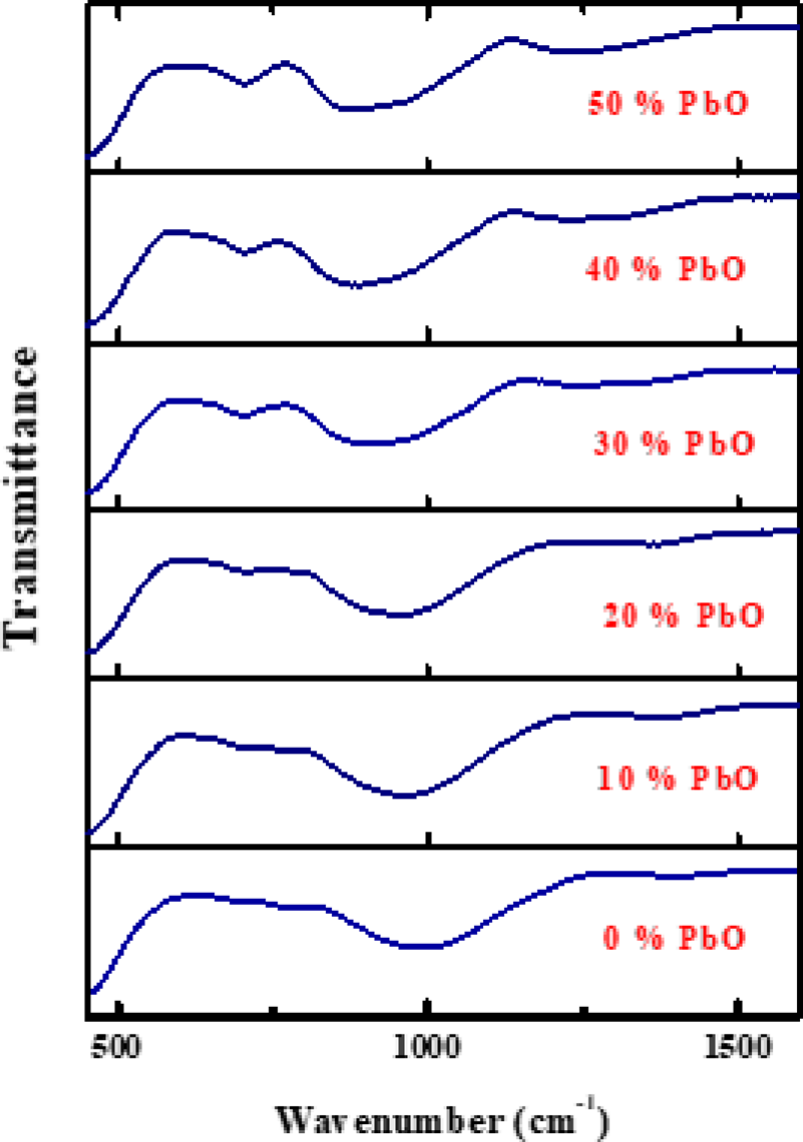
FTIR spectra of the fabricated glass.

The FTIR spectra of the prepared NGS (Figs. 1, 2) reveal important insights into the structural evolution of the glass network as a function of PbO content. The introduction of PbO induces distinct vibrational features associated with Pb–O–Si and Pb–O–B linkages, which are predominantly observed as absorption bands below 600 cm⁻¹. These bands are attributed to bending and stretching vibrations involving heavy metal–oxygen bonds and are common for glass systems containing modifying cations such as Na⁺, Ca²⁺, Mg²⁺, and Al³⁺.

In the mid-infrared range (450–1600 cm⁻¹), multiple significant absorption bands indicative of borate and silicate glass structures were seen. Three significant absorption bands were seen at approximately 707, 970, and 1385 cm⁻¹. The band at around 1385 cm⁻¹, comprising distinct peaks at 1240, 1270, 1310, and 1370 cm⁻¹, is ascribed to the asymmetric stretching vibrations of B–O bonds in trigonal BO₃ units^11,14,34,46–48^. The intensity of this band notably escalates with increasing PbO concentration, reaching a maximum at 50 mol%, signifying an augmentation in Non-Bridging Oxygen (NBO) content. This pattern indicates a partial transformation of BO₄ tetrahedral units into BO₃ units at elevated PbO concentrations, along with the emergence of metaborate species and heightened network depolymerization in the RBPb0–RBPb50 sample series^44^.

Stretching vibrations related to B–O–Si bridge links were detected between 800 and 1260 cm⁻¹, with distinct absorption peaks at 970, 960, 950, 910, 890, and 880 cm⁻¹. The incorporation of PbO up to 20 mol% has a negligible effect on these bands. Nevertheless, when PbO concentrations surpass 30 mol%, a significant redshift of the [BO₄] band is observed, shifting from 945 cm⁻¹ to 931 cm⁻¹, which signifies structural reconfiguration and an augmented generation of NBO resulting from the disintegration of the borate network^11,33,34^. Further evidence of network alteration is shown by the B–O–B stretching vibrations, which are observed in the 620–770 cm⁻¹ region. The peak about 700 cm⁻¹ exhibits heightened intensity with increasing PbO content, while its position stays largely consistent across all compositions. This behavior suggests an increase in borate linkages consistent with the depolymerization of the network and reorganization of boron coordination^31,47^.

As well as the density rises from ~ 2.47 gcm⁻³ for 0% PbO up to 5.29 gcm⁻³ for 50% PbO and is attributed to the high atomic mass and density of PbO (lead oxide is much denser than the base silicate matrix). Therefore, PbO introduces heavier lead atoms into the glass structure, thereby increasing the overall mass density of the material. Simultaneously, this density escalates in relation to radiation shielding; more density typically improves gamma attenuation, since a denser material has a higher number of atoms per volume to engage with photons. The enhanced shielding efficacy at elevated PbO concentrations is partially attributable to this density effect, in conjunction with the increased atomic number of Pb. FTIR research demonstrates that elevated PbO presence leads to substantial alterations in the glass structure, encompassing the emergence of NBO atoms, the transformation between BO₃ and BO₄ units, and modifications in B–O–Si and B–O–B bonding configurations. The structural alterations directly affect the physical qualities and radiation shielding characteristics of the NGS glass system. The discovered spectrum characteristics align well with previously documented patterns and enhance the understanding of lead oxide’s influence on borate–silicate glass networks.

### Mechanical properties

**Fig. 3:**
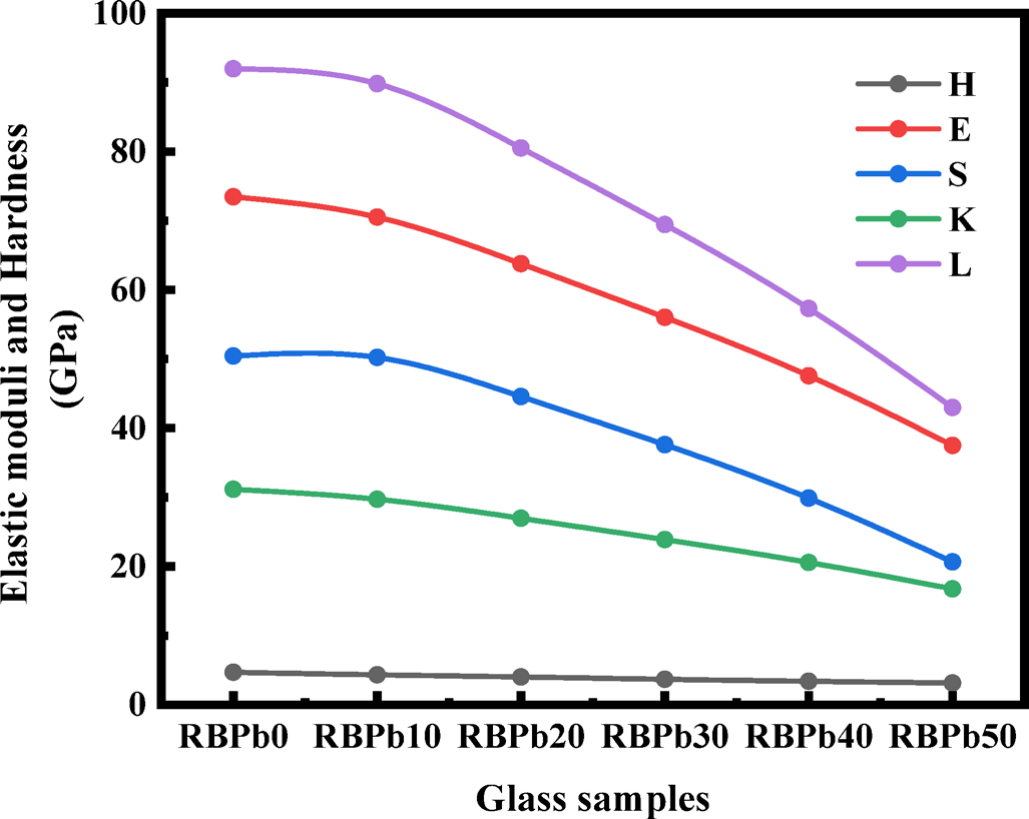
Variation of the elastic moduli (E, S, K, L) and hardness for investigated glass samples.

The addition of PbO to the borosilicate glass system causes significant structural changes that directly affect its mechanical properties. Table 2 provides a detailed summary of the assessed mechanical properties, encompassing elastic moduli (Young’s, shear, bulk, and longitudinal), Poisson’s ratio, and hardness. A gradual reduction in mechanical stiffness is noted with elevated PbO concentration. Young’s modulus diminishes from 73.446 GPa (RBPb0) to 37.480 GPa (RBPb50), whereas the shear modulus declines from 31.162 GPa to 16.749 GPa, as illustrated in Fig. 3. These reductions indicate the softening of the glass matrix resulting from network depolymerization, aligning with the function of PbO as a network modifier. At elevated concentrations, PbO interferes with the glass network by severing Si–O–Si and B–O–B bonds, resulting in the formation of NBO atoms. This leads to a decrease in cross-linking density and an increase in structural disorder, both of which contribute to the noted drop in elastic stiffness and resistance to deformation.

**Table 2:** Variation of elastic moduli, Poisson’s ratio, and hardness of the investigated glass samples.

Mechanical parameters							
Sample code	Packing density	Poisson’s ratio	Hardness (H)	Young’s (E)	Bulk (K)	Shear (S)	Longitudinal (L)
	(V_t_)	(σ)	GPa				
RBPb0	0.573	0.258	4.709	73.446	50.423	31.162	91.973
RBPb10	0.595	0.266	4.329	70.521	50.223	29.701	89.825
RBPb20	0.584	0.262	4.004	63.776	44.566	26.962	80.516
RBPb30	0.560	0.252	3.692	56.031	37.596	23.887	69.447
RBPb40	0.524	0.235	3.395	47.567	29.870	20.577	57.307
RBPb50	0.460	0.198	3.141	37.480	20.662	16.749	42.994

**Fig. 4:**
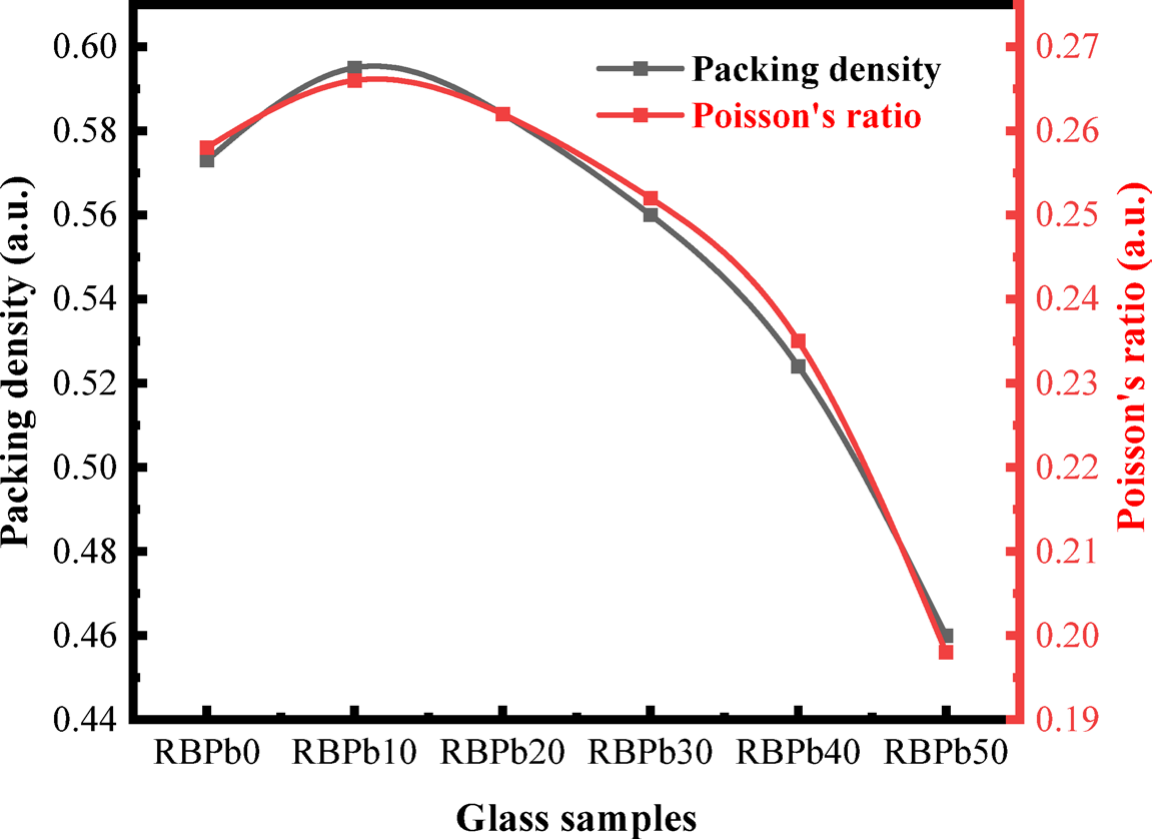
The variation of Poisson’s ratio and packing density for investigated glass samples.

Poisson’s ratio, indicating the network’s stiffness and bonding characteristics, varies from 0.258 (RBPb0) to a maximum of 0.266 (RBPb10), thereafter declining to 0.198 (RBPb50). Generally, elevated Poisson’s ratios (0.3–0.5) indicate diminished rigidity and increased flexibility, whereas reduced values (0.1–0.2) denote stronger bonding and augmented stiffness^34^. The peak at RBPb10 indicates an initial densification and tighter bonding, likely due to partial network modification by PbO. However, further PbO addition promotes NBO formation, reducing bond connectivity and leading to a decline in Poisson’s ratio mirroring the trend observed in packing density (Fig. 4). The peak at RBPb10 signifies beginning densification and enhanced bonding, presumably resulting from partial network alteration by PbO. Nonetheless, further PbO incorporation facilitates NBO formation, diminishing bond connectivity and resulting in a decrease in Poisson’s ratio, paralleling the trend noted in packing density (Fig. 4).

The bulk modulus (K) and longitudinal modulus (L) display a non-monotonic trend, with slight variations noted at intermediate PbO concentrations (Fig. 3). This behavior can be ascribed to the dual function of PbO: at lower concentrations, PbO acts predominantly as a network modifier, disturbing the glass matrix, while at elevated levels, it may partially serve as a glass former, assimilating into the network structure^2,3^. However, the overarching trend substantiates the progressive softening and structural depolymerization of the glass network as PbO content increases. Hardness (H), a crucial metric reflecting a material’s resistance to indentation and abrasion^48^, exhibits a decreasing trend, diminishing from 4.709 GPa (RBPb0) to 3.141 GPa (RBPb50) as indicated in Table 2. This reduction corresponds with the rise in NBOs and diminished network connectivity, both of which lead to a more compliant and less scratch-resistant structure.

The incorporation of PbO results in a consistent decline in mechanical properties, such as stiffness, hardness, and elasticity, attributable to its activity as a network modifier and its influence on the depolymerization of glass structure. The results indicate that, instead of a direct enhancement in mechanical performance, mechanical stiffness diminishes while the glass retains satisfactory structural integrity. This equilibrium is especially significant when evaluating the superior radiation shielding properties imparted by the high atomic number and density of PbO, hence augmenting the multifunctionality of the engineered glass system.

### Radiation shielding parameters

#### EBF and EABF

**Fig. 5:**
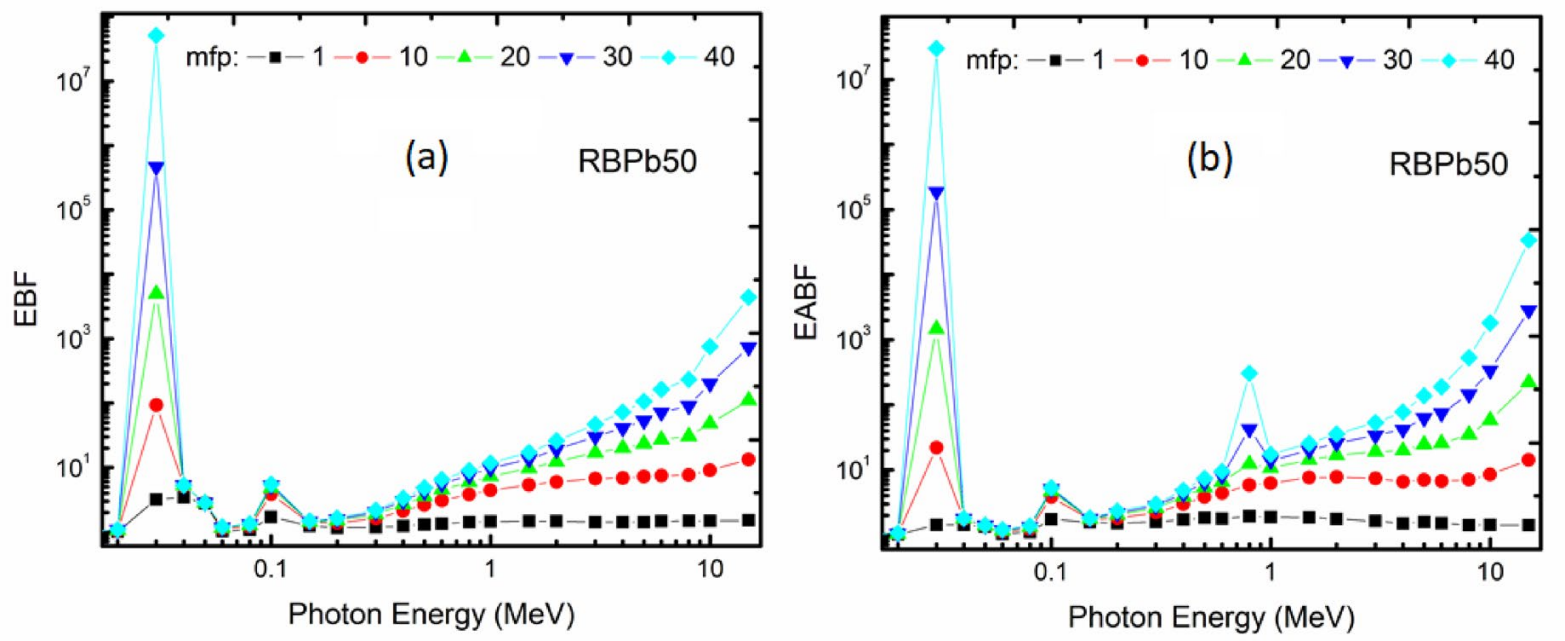
Correlation influence of photon energy for RBPb50 values **a** EBF and **b** EABF.

Both EABF and EBF results were calculated as penetration multifunctional by depth (mfp) for all the glass systems prepared across energy windows of (0.015 to 15 MeV), as illustrated in Fig. 5. In general, both EBF and EABF exhibited an increasing trend with increasing penetration depth, and the impact of varying penetration depth was found to be energy dependent. The BF for each radiation exposure and absorption exhibited the lowest values at low energy levels (0.1 MeV), signifying the predominance of the photoelectric effect. A significant peak at roughly 0.02 MeV was detected, attributable to the L-absorption edge of Pb, consistently present in all glass samples. A further peak corresponding to the K-absorption edge of Pb was observed at 0.1 MeV, along with the heightened concentration of Pb^47,48^.

As the energy level ascended to the intermediate range (0.1 to 10 MeV), both EBF and EABF exhibited an upward trend, attaining their maxima at 1.5 MeV. This discovery corresponds with the anticipated impact of the Compton scattering effect, recognized as substantial at intermediate energy levels. At elevated energy levels (> 10 MeV), the occurrence of pair production consistently increased with rising incident energy. The new findings agree with earlier published studies elsewhere^29,47^.

#### Proton and alpha shielding properties

**Fig. 6:**
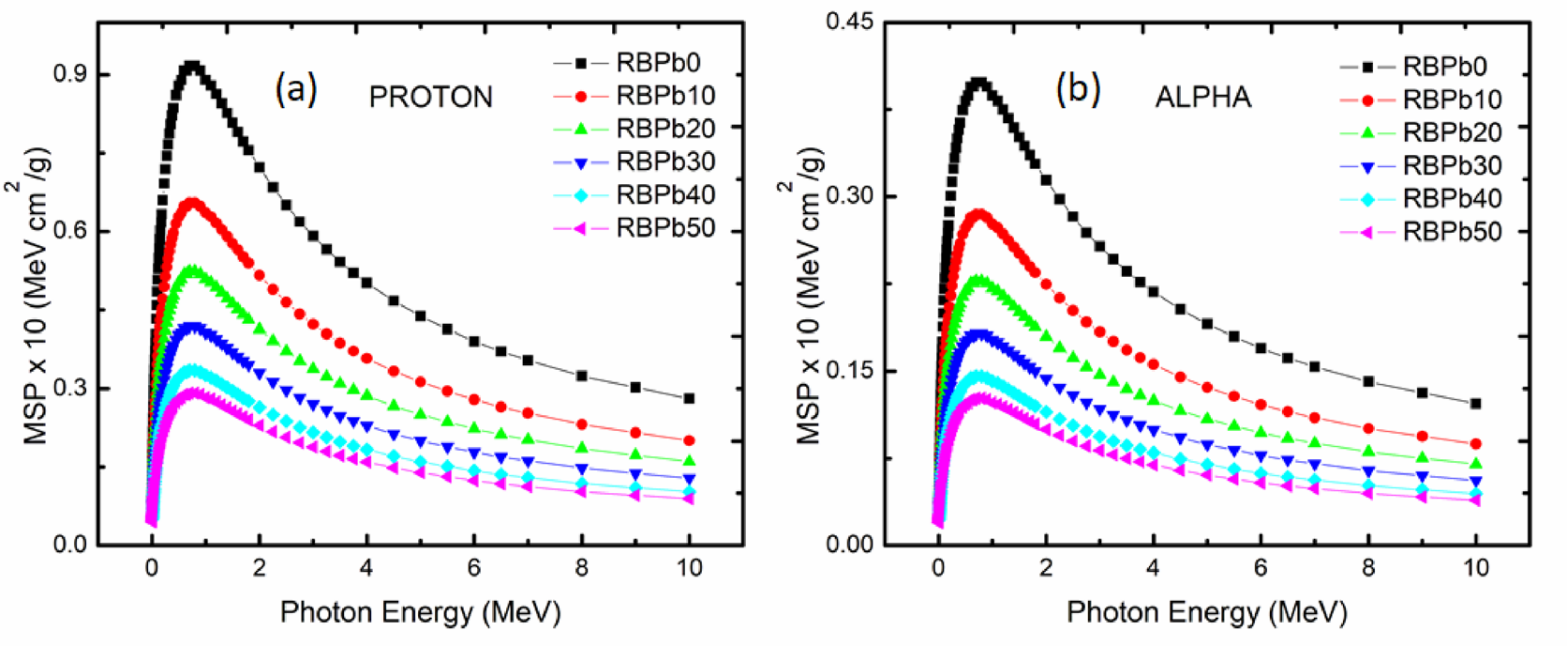
Mass stopping power for Proton (**a**) and Alpha (**b**) shielding properties of prepared glasses system correlation to kinetic energy.

**Fig. 7:**
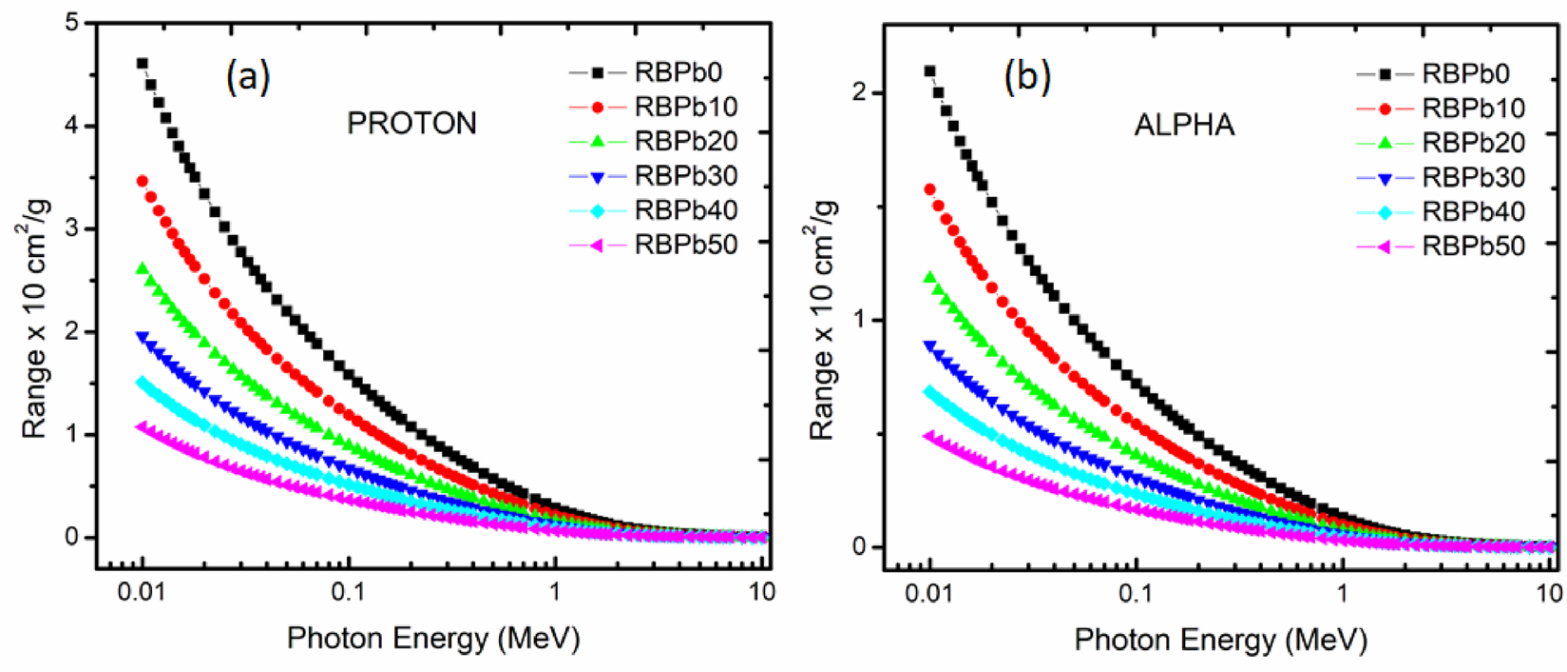
Projectile range for Proton (**a**) and Alpha (**b**) shielding properties of prepared glasses system correlation to kinetic energy.

The values of the prepared glasses’ proton and alpha Mass Stopping Power (MSP) are shown in Fig. 6. As can be seen in figures (a) and (b), both proton and alpha values rose as kinetic energy (up 1 MeV) increased. The MSP values drastically decreased in the energy window of 1–2 MeV, and the decline persisted at higher energies (> 2 MeV), albeit at a slow rate. The samples with the lowest proton and alpha MSP values were those with the highest Pb concentration (RBPb50). The “projected range” terminology refers to the predicted track chasm to which an alpha or proton will pierce until rests in an absorber material^43,47,48^. The estimated alpha and proton ranges in the current investigation were likewise established using the SRIM code^26–28,31,43^. The samples produced, particularly RBPb50, acquired low projected range values, as shown in Fig. 7a and b.

#### Specific absorbed fraction of energy (SAFE)

**Fig. 8:**
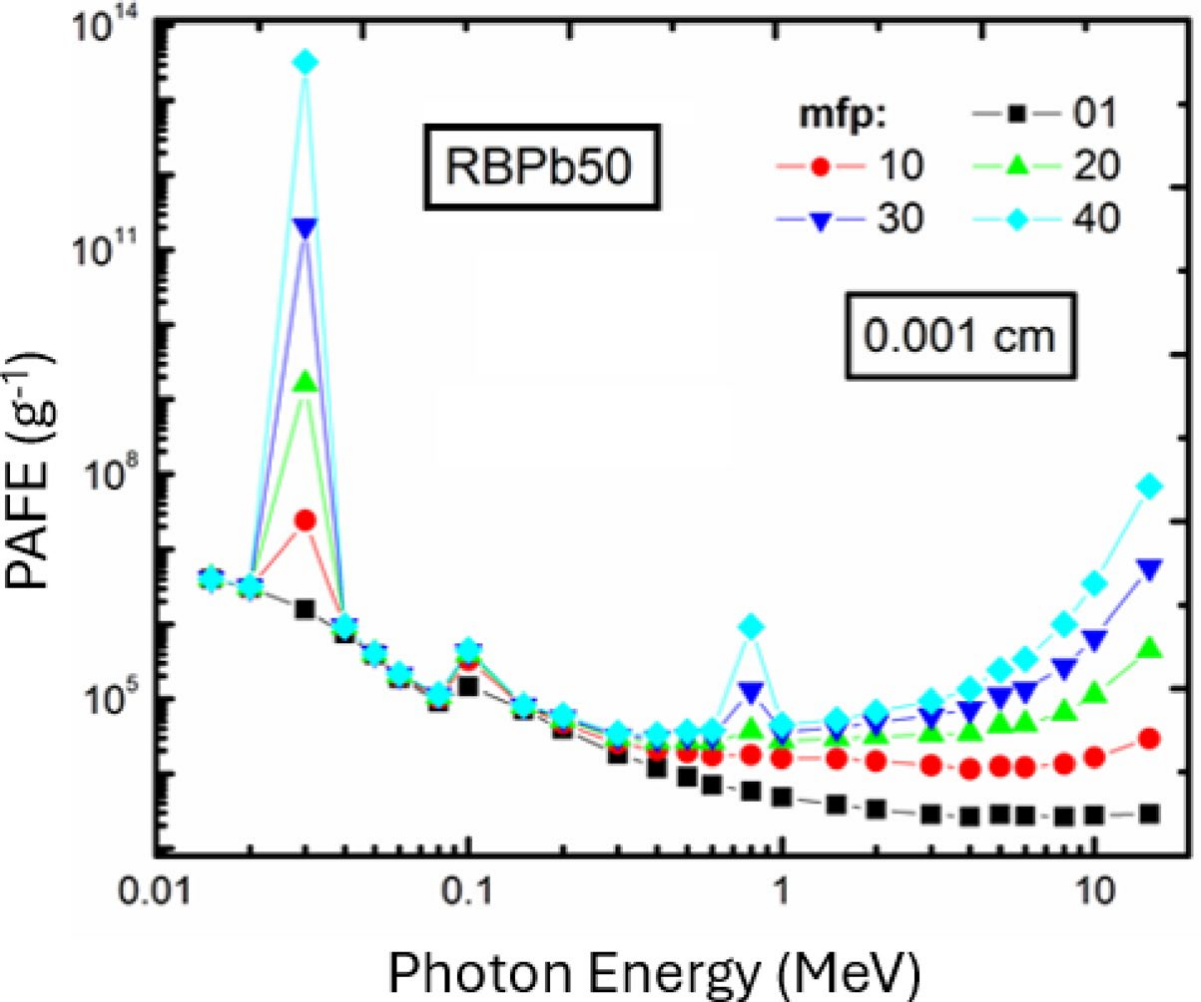
The values of PAFE for RBPb50 at 0.001 cm in different mfps.

Figure 8 presents the Specific Absorbed Fraction of Energy (SAFE) for RBPb50 glass up to 40 mean free paths (mfp) at a thickness of 0.001 cm. The data obtained indicates that the SAFE values rise until they reach approximately 0.4 MeV, after which they decrease that related to the interaction of photoelectric interaction and the Compton scattering coefficients, which are directly affected by the density and chemical composition of the samples. Approximately 0.4 MeV, the photoelectric and Compton scattering coefficients leading to the peak values of SAFE.

The dominance of Compton scattering makes more scattering which leads to partial absorption but at low levels of energy, the values of SAFE decrease due to the prevalence of the photoelectric effect. Conversely, at high energy levels, characterized by pair creation phenomena, the SAFE values decrease again due to the diminished likelihood of specific absorption^48^. The current result demonstrated valuable value of SAGE and energy-dependent behavior as well as its relation to glass composition, density, and photon interactions.

**Fig. 9:**
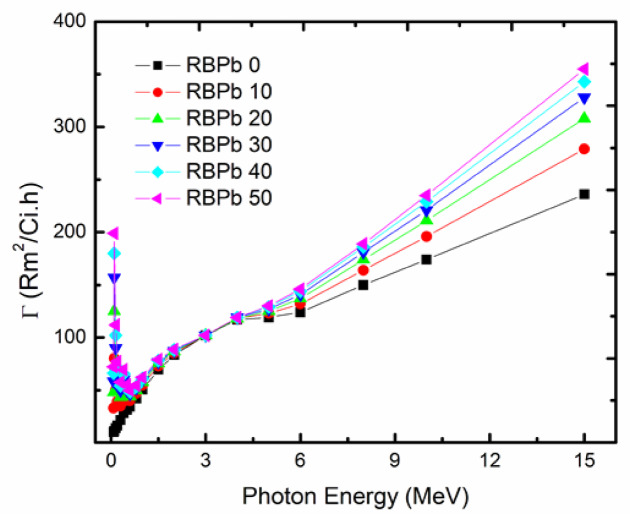
Index of gamma-rays (Γ) for the glasses at various energy levels.

Gamma-rays dose deposition in materials can be estimated using specific gamma-rays constants (Γ), which can be determined for different materials. The recycling glass samples in Fig. 9 show the trend of Γ values consistent across all glasses. The highest Γ values were observed for RBPb50, with values of 263, 259, 254, and 249 at 15 MeV for RBPb50, RBPb40, RBPb30, RBPb20, RBPb10, and RBPb0, respectively. High and low energy levels correspond to high Γ values due to dominant photoelectric (PE) and pair production (PP) interactions, which are affected by atomic number. Conversely, the Γ values are low at medium energy levels because of Compton scattering (CS) and an increase in scattered photons. The appearance of an edge at 88 keV can be attributed to Pb.

**Fig. 10:**
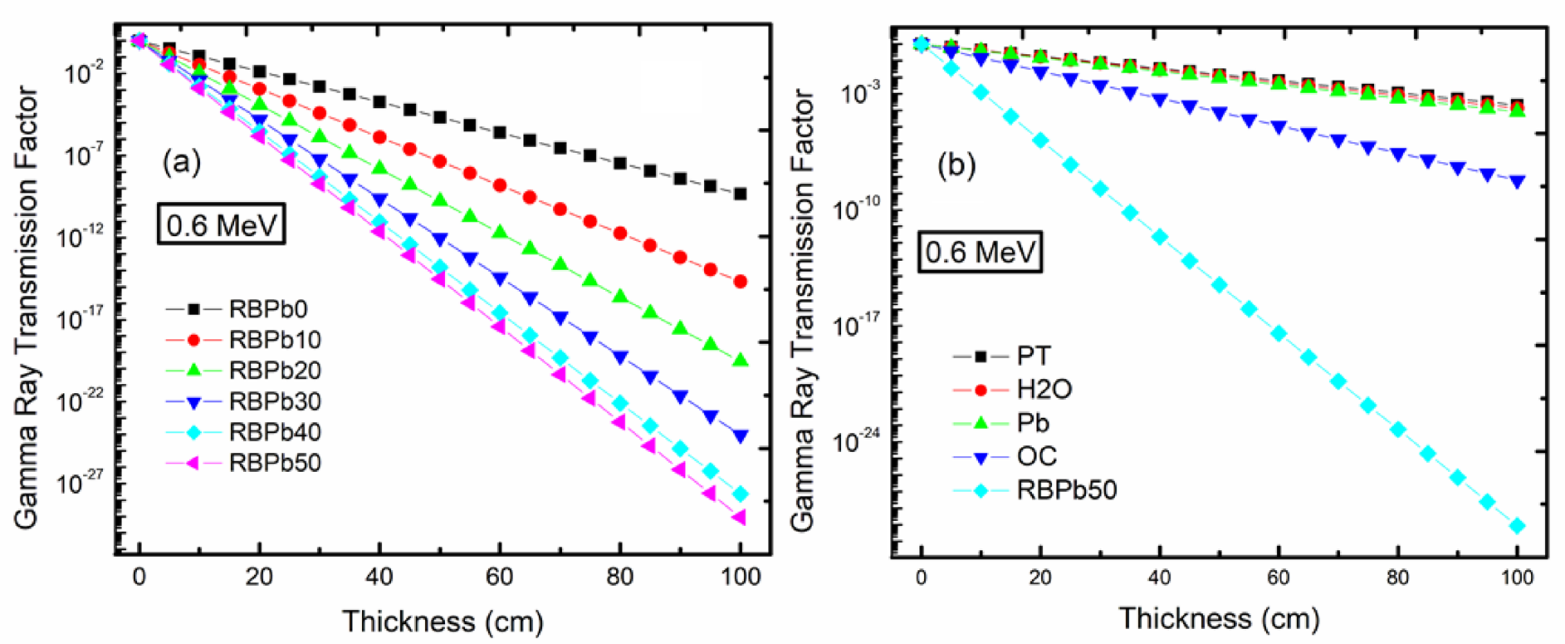
Gamma-rays Transmission Factor (TF) at different thicknesses in the photon energy range 0.6 MeV of the new glasses (**a**) and compared with some shielding materials (**b**). [P-T: Polythene; Pb: Poly-boron; O-C: Original Concrete].

The variation of the GTF is seen in Fig. 10, showing how the glass thickness directly affects TF values. For the selected glasses, TF values rapidly decline with increasing glass sample thickness and rise with increasing photon energy. The outcome suggests that as the PbO concentration rises, so does the gradient of the glass samples. The glass sample that contributes the most PbO (RBPb50) compared to other glass samples has the least amount of gradient in all energy values. The interaction between Pb atoms in glass samples and gamma radiation will increase as glasses are enriched with a high atomic number of Pb atoms. This implies that more photon energy must be absorbed in order for Pb atoms to eject electrons. When photon and Pb atom interactions in glasses increase, the amount of absorbed gamma through the glass also increases, which raises values.

**Fig. 11:**
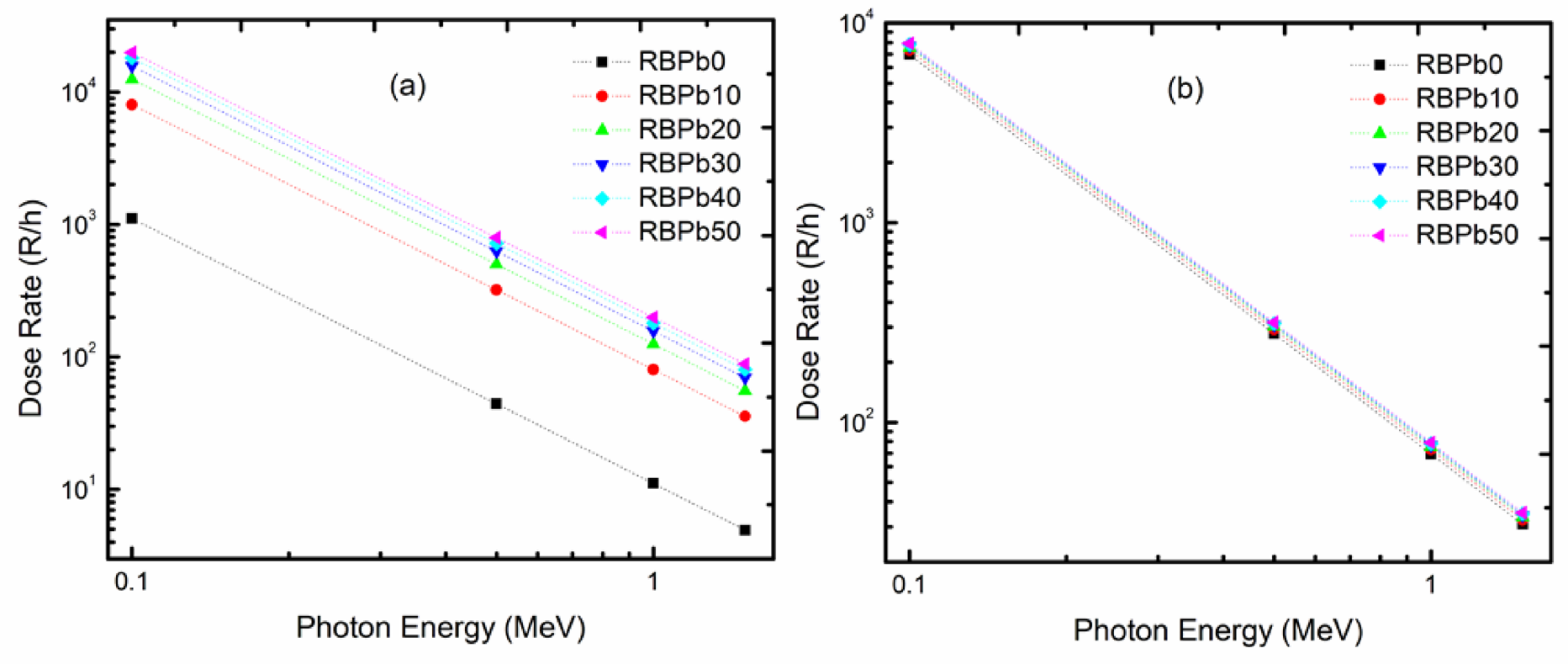
Variations of gamma dose rate (R/h) at different distances from the source for the prepared glasses.

The results shown in Fig. 10b demonstrate that the GTF for these sources decreases in the following order: RBPb50 < regular concrete < poly-boron < water < polyethylene. Therefore, RBPb50 glass, which has the highest Pb content (low TF), will be the best shielding material for these gamma sources, whereas polyethylene has the lowest place in terms of thickness. Figure 11 illustrates the fluctuations in the dosage rate (R/h) from a gamma-rays source at various prepared glass thicknesses. The gamma-rays dosage rate falls as the medium’s thickness increases in all prepared samples. Additionally, we observed a rise in PbO content (0 to 50 mol%). Low energy (0.1 MeV) makes the differences between glass samples obvious, whereas high energy (> 1 MeV) gradually ignores these differences. The absorber’s composition and even thickness have little discernible impact at high energy levels.

The future studies will include experimental measurements to validate the model’s predictions. This clarification and additional context make it clear that the current mechanical property results are theoretical and highlight that our conclusions about tunable stiffness are drawn from modeled data, pending further experimental investigation and radioanalytical confirmation.

## Conclusion

The investigation of Novel Glass Systems (NGS) derived from Solid Waste Resources (SWR) revealed promising structural, mechanical, and radiation-shielding characteristics. The incorporation of PbO into the glass matrix produced a systematic modification of the mechanical properties, as evidenced by a decrease in Young’s modulus from 73.446 to 37.480 GPa and a reduction in hardness from 4.709 to 3.141 GPa as the PbO content increased in steps of 10 mol% up to 50 mol%. These trends indicate a progressive reduction in glass rigidity and scratch resistance, which can be attributed to structural depolymerization within the glass network. Structural changes induced by PbO addition were further confirmed by FTIR spectroscopy, which revealed notable variations in vibrational bands and peak intensities. The spectrum alterations indicate the emergence of Non-Bridging Oxygen (NBO) species linked to boron–oxygen units, resulting in various bonding topologies within the glass network. Such structural rearrangements provide insight into the role of PbO as a network modifier and support the suitability of the developed glass system for safety-oriented and functional applications. The radiation-shielding performance of the fabricated samples was found to be outstanding. The Exposure Buildup Factor (EBF) and Energy Absorption Buildup Factor (EABF) exhibited pronounced energy-dependent behavior, with distinct maxim at specific penetration depths, indicating effective attenuation across a wide range of gamma-rays energies. Additionally, the Specific Absorbed Fraction of Energy (SAFE) and Gamma-rays Transmission Factor (GTF) analyses confirmed the strong shielding capability of the prepared glasses. The composition with the greatest PbO concentration (RBPb50) exhibited improved shielding performance, underscoring the essential function of PbO in improving gamma-rays attenuation. The findings highlight the considerable potential of the new PbO-rich glass technology for practical radiation-shielding applications. The effective use of solid waste-derived materials, such as Spent Lead-acid Batteries (SLB) and Vehicle Glass (VG), exemplifies a sustainable method for producing shielding materials, aiding waste valorizations, environmental conservation, and the promotion of circular economy initiatives.

## Data Availability

All results for the present study are included in this published version of article. The analyzed data is free available and would be requested on reasonable correspondence.
